# Direct superior approach versus posterolateral approach in total hip arthroplasty: a randomized controlled trial on early outcomes on gait, risk of fall, clinical and self-reported measurements

**DOI:** 10.1080/17453674.2020.1865633

**Published:** 2021-01-07

**Authors:** Michele Ulivi, Luca Orlandini, Jacopo A Vitale, Valentina Meroni, Lorenzo Prandoni, Laura Mangiavini, Nicolò Rossi, Giuseppe M Peretti

**Affiliations:** a IRCCS Istituto Ortopedico Galeazzi, Milan;; b Residency Program in Orthopedics and Traumatology, University of Milan, Milan;; c Department of Biomedical Sciences for Health, University of Milan, Milan, Italy

## Abstract

Background and purpose — Several surgical approaches are used in primary total hip arthroplasty (THA). In this randomized controlled trial we compared gait, risk of fall, self-reported and clinical measurements between subjects after direct superior approach (DSA) versus posterolateral approach (PL) for THA.

Patients and methods — Participants with DSA (n = 22; age 74 [SD 8.9]) and PL (n = 23; age 72 [7.7]) underwent gait analysis, risk of fall assessment and Timed Up and Go Test (TUG) before (PRE), 1 month (T1) and 3 months after (T3) surgery. Data on bleeding and surgical time was collected.

Results — DSA resulted in longer surgical times (90 [14] vs. 77 [20] min) but lower blood loss (149 [66] vs. 225 [125] mL) than PL. DSA had lower risk of fall at T3 compared with T1 and higher TUG scores at T3 compared with T1 and PRE. PL improved balance at T3 compared with T1 and PRE. Spatiotemporal gait parameters improved over time for both DSA and PL with no inter-group differences, whereas DSA, regarding hip rotation range of motion, showed lower values at T3 and T1 compared with PRE and, furthermore, this group had lower values at T1 and T3 compared with PL. All foregoing comparisons are statistically signficant (p < 0.05)

Interpretation — DSA showed longer surgical time and lower blood loss compared with PL and early improvements in TUG, spatiotemporal, and kinematic gait parameters, highlighting rapid muscle strength recovery.

The lateral and posterolateral surgical approaches to the hip, either performed traditionally or with a mini invasive approach, foresee incision of the iliotibial band and imply the incision of the abductor muscles or the external rotator muscles (Murphy and Millis 1999). While the impact of the damage of these muscles on patients’ outcome is well known, the effect of surgical technique on the stabilization system, such as the fascia lata and the entire fascial system, has rarely been studied. It is known that a distortion within the fascial system leads to a loss of function and changes in the connecting structure of the locomotor system, as the fascial system is the key for stability and sensorimotor function (Huijing 2012, Vitale et al. 2019). The minimally invasive direct superior approach (DSA) combines minimal invasiveness and the advantage of preserving the fascia lata and the abductor muscles. DSA was first described by Stephen Murphy: it consists of a blunt dissection of the gluteus maximus muscle and a superior capsulotomy to reach the femoral neck and requires specialized instrumentation to preserve the tendons of the extrarotator muscles and to easily reach the femoral neck (Murphy and Millis 1999).

This randomized controlled trial (RCT) investigates whether a modified DSA with avoidance of sectioning the iliotibial band (ITB) can elicit better early outcomes in gait and risk of fall. Total hip arthroplasty (THA) was performed with the aid of dedicated and modified instrumentation. We hypothesized that DSA, with the preservation of the ITB, would lead to a faster recovery compared with the posterolateral approach (PL).

## Patients and methods

### Participants and design

**Figure 1.**
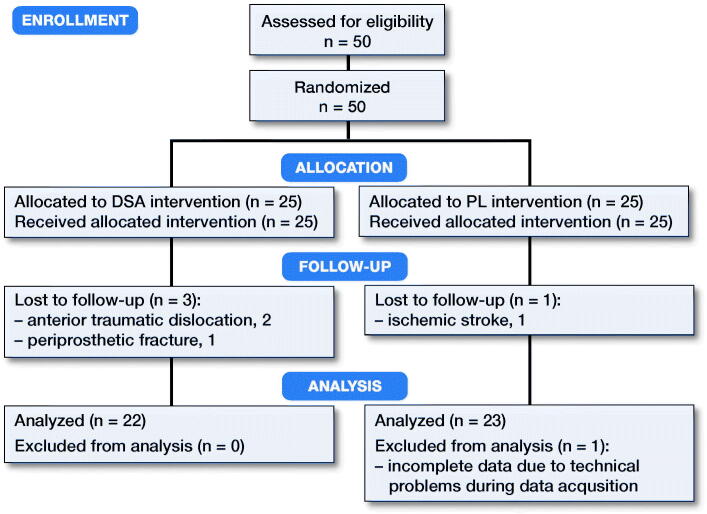
CONSORT 2010 flow diagram of steps involved in the screening and enrollment of the DSA and PL groups. PL, posterolateral mini approach; DSA, direct superior approach.

Based on the literature, we considered it clinically significant to observe a mean effect size of 0.9 in the hip rotation range of motion (ROM) between the 2 groups. Therefore, considering an α level with p = 0.05 and a power of 90%, 22 subjects would be necessary in each of the 2 groups to detect a statistically significant difference in hip rotation ROM. To prevent possible dropout of subjects during the study (estimated to 10–15%), the sample size was increased to a total of 50 subjects. The inclusion criteria were presence of noninflammatory degenerative joint disease, rheumatoid arthritis, age between 60 and 75, BMI between 18 and 30 and absence of contralateral THA (Figure 1). The subjects were randomly allocated to one of the 2 treatment groups with a computer-generated 1:1 randomization list.

### Surgical procedure

Between April 2017 and December 2018 1 senior orthopedic surgeon (MU), experienced in the PL approach, performed all surgeries. All patients were positioned on the lateral decubitus of the contralateral side. In the PL group a mini standard approach was used. The total incision of the fascia lata accounted for approximately 3–5 cm proximally and distally to the tip of the greater trochanter. In the DSA group, no iliotibial band section was performed; however, splitting of the gluteus maximus muscle, short external rotator preservation with selective division of the piriformis tendon and a posterior capsulotomy were performed. Implants for both DSA and PL were the Accolade II femoral stem (Stryker, Michigan, USA) and Trident cup with poly insert (Stryker, Michigan, USA). Surgical time, blood loss, pre- and post-surgery hemoglobin levels, and adverse outcomes were recorded. Radiographs were obtained accordingly to routine procedures preoperatively, postoperatively, and at 3 months and 6 months.

### Self-reported and clinical outcome measures

All patients underwent preoperatively (PRE), at 1 week (W1), 2 weeks (W2), 3 weeks (W3), 1 month (T1), 3 months (T3) and 6 months (T6) after surgery the following clinical evaluations: the Hip Disability and Osteoarthritis Outcome Score (HOOS) (Klässbo et al. 2003), subjective ratings of pain by VAS, the Harris Hip Score (HHS), and the SF-12. Evaluation of all scales at T6 was conducted by the same investigators (VM, LP and NR).

### Gait analysis

Patients underwent clinical gait analysis before (PRE), 1 month (T1), and 3 months after (T3) surgery in the Motion Analysis Laboratory of our institute. For the gait analysis, a Helen Hayes marker set of 22 retro-reflective passive markers was used and a Davis biomechanical model was applied during data acquisition and processing (Davis et al. 1991). Patients were asked to walk as best as they could at a self-selected speed without walking aids along a 13-meter walkway at least 6 times. An optoelectronic system (SMART-D, BTS Bioengineering, Milan, Italy) with 8 infrared cameras (sampling rate 100 Hz) was used for spatiotemporal and kinematic data acquisition. Mark trajectories were recorded, reconstructed, and processed by SMART-D Analyzer software (BTS Bioengineering, Quincy, MA, USA). The gait parameters were: (1) spatiotemporal variables: stance phase (percentage), swing phase (percentage) step length (meters), stride length (meters), gait speed (m/s), and gait cadence (steps/minute); stance and swing were normalized as a percentage of the gait cycle; (2) kinematic parameters (in degrees): hip flexion–extension ROM, hip abduction–adduction ROM, hip rotation ROM, hip obliquity ROM.

### Risk of fall assessment and Timed Up and Go test

The risk of fall was evaluated before (PRE), 1 month (T1), and 3 months after (T3) surgery with the OAK system (Khymeia, Padova, Italy). For this purpose, the device provides an automated version of the Brief-BESTest (Padgett et al. 2012). It yields a point-score from 0 to 24 and it has been shown that the relative optimal cutoff point was a 16 point score out of 24; a point score between 17 and 24 classifies a subject as low risk who would otherwise be classified as being at medium/high risk (Castellini et al. 2019). In addition, the Timed Up and Go test (TUG) (Podsiadlo and Richardson 1991) was performed at PRE, T1, and T3.

### Statistics

#### Baseline characteristics

Unpaired Student’s t-tests, or non-parametric Mann–Whitney rank tests when needed, were used to test the differences between groups. Fisher’s exact test was used to evaluate the differences in the frequency distribution of females and males in the 2 study groups while intragroup (pre- vs. post-surgery) and intergroup (DSA vs. PL) differences in hemoglobin levels were checked using 2-way analysis of variance (ANOVA) with Bonferroni’s multiple comparisons test. P < 0.05 was considered statistically significant (Table 1).

**Table 1.** Baseline characteristics of DSA and PL. Data are mean (SD)

Factor	Approach	
	direct superior	posterolateral
	(n = 22)	(n = 23)
Age (years)	74 (8.9)	72 (7.7)
Height (m)	173 (5.2)	174 (6.4)
Weight (kg)	69 (10)	72 (11)
BMI	23 (2.8)	24 (2.0)
Sex (Male:Female)	7:15	10:13
Gait speed at PRE (m/s)	0.60 (0.25)	0.55 (0.22)
Surgical time (minutes)	90 (14)	77 (20) **^b^**
Bleeding (mL)	149 (66)	225 (125) **^a^**
Hemoglobin (g/dL)		
preoperative	14 (1.2)	14 (0.79)
postoperative	12 (1.2)	12 (1.0)

**^a^**p = 0.04; **^b^** p = 0.002 (Mann–Whitney rank test due to non-normally distributed data).

#### Gait and risk of fall

Gait parameters, OAK, and TUG data were acquired at 3 time points, PRE, T1, and T3 (before, 1 month, and 3 months after surgery), for each group (DSA and PL). The normal distribution of each parameter was then checked with the Shapiro–Wilk test. The non-normal distributed variables were tested using non-parametric methods. Mixed ANOVA was applied to each gait parameter, OAK and TUG data. 1st, we checked the presence of an interaction between the 2 factors, within-subjects factor (time) and between-subjects factor (groups). 2nd, we evaluated the simple main effects of group and time. The time effect was assessed using 2 separate one-way repeated-measure ANOVAs (1 for DSA and 1 for PL) followed by the Tukey–Kramer post-hoc test for differences in each parameter between PRE, T1, and T3. The effect of group was determined by evaluating the differences in each parameter between the DSA and PL with 3 separate unpaired Student’s t-tests (one for PRE, T1, and T3). Significance was set at p < 0.05.

#### Self-reported clinical measures

HOOS, VAS, HHS, and SF-12 data were acquired at 7 time points (PRE, W1, W2, W3, T1, T3, T6). The normal distribution of each measure was checked with the Shapiro–Wilk test. Mixed ANOVA was applied to all data and the non-normal distributed variables were tested using non-parametric methods. Significance was set at p < 0.05. All statistical analysis was performed using GraphPad Prism version 6.00 (GraphPad Software, San Diego, CA, USA).

#### Ethics, registration, funding, and potential conflicts of interest

The study was supported by the Italian Ministry of Health (Ricerca Corrente) and was approved by the Ethical Committee of Vita-Salute San Raffaele University (Milan, Italy; registration number: 129/INT/2016) in compliance with current national and international laws and regulations governing the use of human subjects (Declaration of Helsinki II). Written informed consent was obtained from all patients. The study was registered at clinicaltrials.gov (NCT04358250). This study has received a liberal grant from Stryker, which has not been involved at any point neither in the conception of study design, collection, analysis, interpretation of the data nor in writing of this manuscript. LO is a paid part-time employee of a manufacturer (Smith&Nephew) while MU, JV, VM, LP, LM, NR and GP declared that they have no conflict of interest.

## Results

45 patients were included in the final analysis, 22 for DSA and 23 for PL (Table 1). The number of major adverse events was higher in DSA (3/25) than in PL (1/25). 1 periprosthetic fracture and 2 anterior dislocations due to falls occurred in DSA and 1 ischemic stroke occurred in the PL group. No other adverse events were observed. The groups were similar regarding height, weight, BMI, age, and sex distribution, whereas DSA had longer surgical times (90 [SD14] minutes vs. 77 [20] minutes; p = 0.002) but lower blood loss (148 [66] mL vs. 225 [125] mL; p = 0.03) than PL.

### Risk of fall and TUG

DSA had higher OAK values at T3 compared with T1 (p = 0.03) and higher TUG scores at T3 compared with T1 (p = 0.009) and PRE (p = 0.009). Furthermore, PL registered greater OAK scores at T3 compared with T1 (p = 0.02) and PRE (p = 0.04) while TUG did not show any statistically significant difference (Figure 2 and Table 2, see Supplementary data).

Figure 2.Mean (dot) and standard deviation (whiskers) of OAK and TUG for DSA group (n = 22) and PL group (n = 23) before (PRE), 1 month (T1), and 3 months (T3) after surgery. Dashed line indicates the 16 cutoff point score for OAK (i.e., a point score below 16 indicates a medium/high risk of fall).

**Figure:**
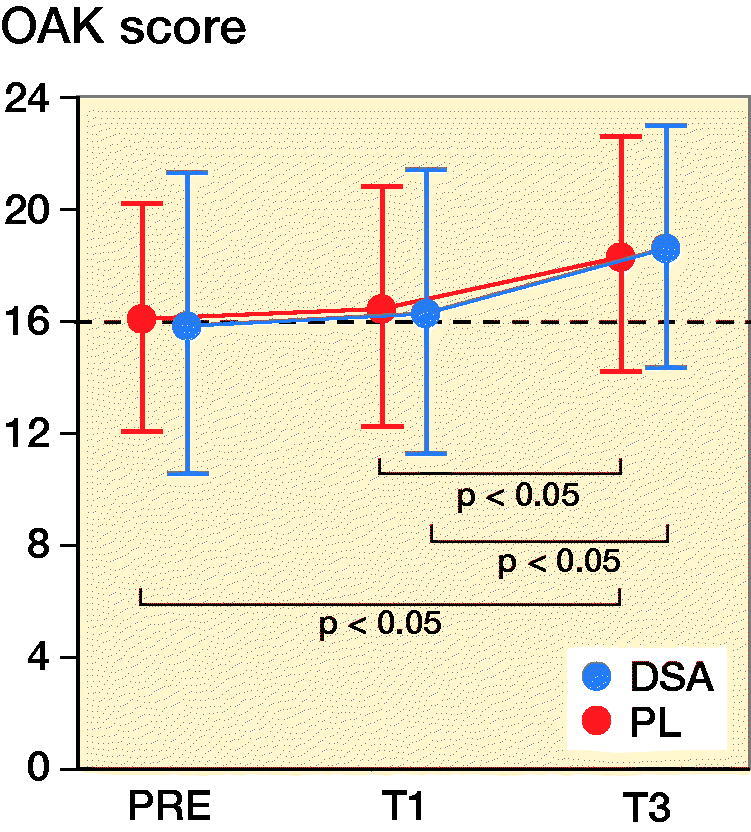

**Figure:**
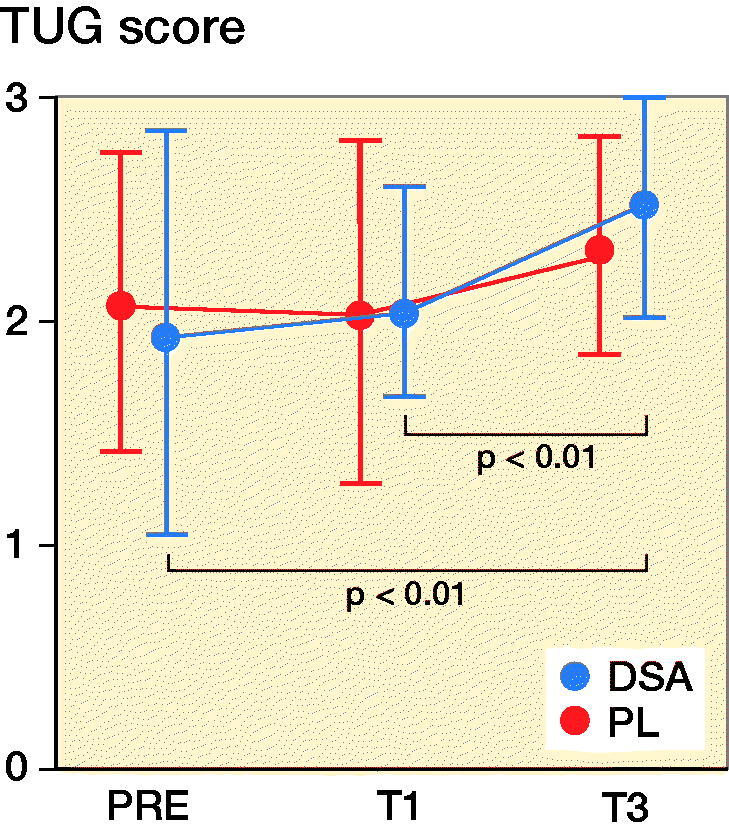

### Spatiotemporal variables

Stance phase and swing phase of the operated limb did not show any statistically significant intra- and inter-group difference whereas both DSA (p = 0.04) and PL (p < 0.001) had lower swing phase and stance phase values in T3 compared with T1. DSA increased step length (+0.05 m for both legs; p = 0.001 and p < 0.001 for operated and non-operated leg respectively) and stride length (+0.10 m for both legs; p = 0.007 and p = 0.001 for operated and non-operated leg respectively) in T3 compared with T1 and, in addition, PL significantly increased from PRE and T1 to T3 for both stride and step length (Figure 3 and Table 2, see Supplementary data). DSA increase gait cadence from 94 [2] steps/minute to 101 [13] steps/minute (p < 0.001) and gait speed from 0.56 [0.23] m/s to 0.73 [0.23] m/s (p < 0.001) from T1 to T3.

Figure 3.Mean (dot) and standard deviation (whiskers) of stance phase, swing phase, step length, stride length, cadence, and velocity for DSA group (n = 22) and PL group (n = 23) before (PRE), 1 month (T1), and 3 months (T3) after surgery.

**Figure:**
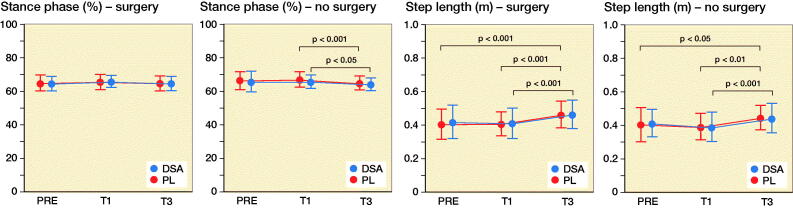

**Figure:**
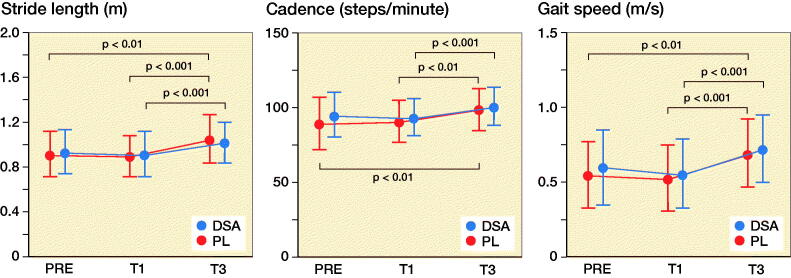

### Kinematic variables

**Figure 5.**
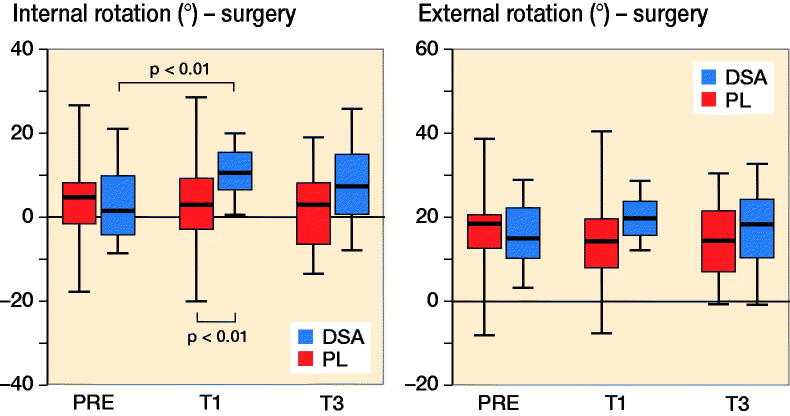
Median (black line), first and third quartiles (box), and minimum and maximum (whiskers) of hip internal and external rotation values for DSA group (n = 22) and PL group (n = 23) before (PRE), 1 month (T1) and 3 months (T3) after surgery.

PL increased hip flexion–extension ROM of the operated leg from PRE (p < 0.001) and T1 (p < 0.001) to T3 while DSA did not show any statistically significant difference. Regarding hip abduction–adduction ROM, DSA registered higher values in T3 compared with PRE (p = 0.03) for the surgical leg, while PL showed an increased from T1 to T3 (p = 0.003) for the healthy leg (Figure 4 and Table 3, see Supplementary data). Hip rotation ROM for the healthy leg did not show any statistically significant inter- or intra-group difference while DSA for the operated limb, showed lower values at T3 (p = 0.01) and T1 (p = 0.005) compared with PRE and, furthermore, had lower values at T1 (p = 0.04) and T3 (p = 0.04) compared with PL (Figure 5). Finally, PL did not show significant differences in hip obliquity ROM while DSA revealed a significant increase only at T1 compared with PRE for both the operated (p = 0.007) and non-operated (p = 0.04) lower limb.

Figure 4.Median (black line), first and third quartiles (box), and minimum and maximum (whiskers) of hip extension–flexion ROM, hip abduction–adduction ROM, hip obliquity ROM, and hip rotation ROM, for DSA group (n = 22) and PL group (n = 23) before (PRE), 1 month (T1) and 3 months (T3) after surgery.

**Figure:**
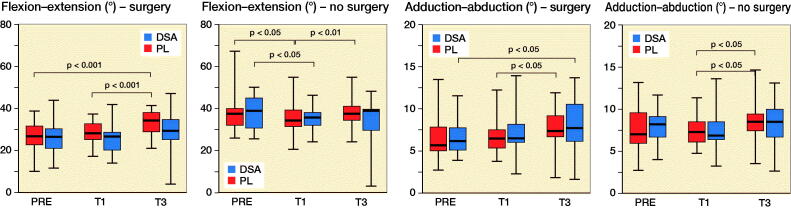

**Figure:**
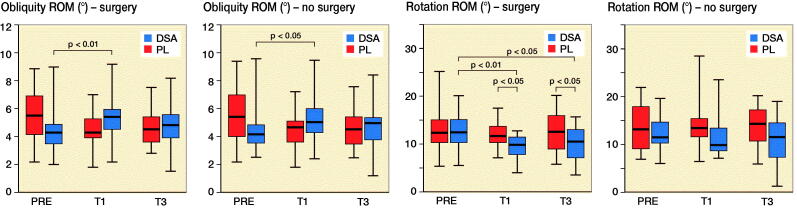

### Self-reported clinical measures

HOOS, HHS, SF-12, and VAS scores improved from PRE to T6 (p < 0.001), while no statistically significant differences between groups were detected. All self-reported measures for both DSA and PL showed a constant improvement from baseline to each evaluation time with a plateau observed at T3.

## Discussion

To our knowledge, this is the first RCT assessing the short-term impact of DSA and PL approaches in total hip arthroplasty. DSA foresees the complete sparing of the fascia lata, whereas in PL this anatomical structure is incised and laterally sutured. We found that DSA had longer surgical time but lower blood loss and, in addition, DSA showed better TUG at T3 while PL did not display any difference over time. Overall, we found similar results between DSA and PL in risk of fall, spatiotemporal, and kinematic gait variables; only hip rotation ROM displayed inter-group differences with lower values at T1 and T3 for DSA compared with PL. Results of HOOS, HHS, SF-12, and VAS were similar between DSA and PL. Thus, our initial hypothesis was partially confirmed.

In a previous study, the consequences of dissection and suturing the fascia lata were studied using MRI and ultrasonography and it was reported that the fascia has strong relationships with the underlying musculature. It appears that an intact fascia represents a vital component for the normal function of thigh muscles and knee control in bipedal locomotion (Huijing 2012). As a consequence, our primary aim was to evaluate 3D movement differences during walking (i.e., gait analysis) between the 2 study groups. The risk of fall, evaluated by the OAK device, did not show any statistically significant inter-group difference but a significant improvement for both DSA and PL was detected at T3 compared with earlier assessments. TUG was significantly different from preoperative values to T1 (p = 0.009) and T3 (p = 0.009) only for DSA but not for PL (Figure 2). However, the inter-group difference was not statistically significant. All spatiotemporal parameters significantly improved in both groups. Gait cadence and speed had a highly significant improvement from T1 to T3, which was more pronounced in the DSA group. The analysis of the kinematic parameters deserves particular attention and accurate interpretation. While ROM improvement for hip flexion/extension was significant in PL from PRE to T3 and from T1 to T3, results on hip abduction/adduction ROM showed a different pattern with a significant difference from PRE to T3 only in the DSA group. However, the inter-group differences for these 2 kinematic parameters were not significant.

It is noteworthy that hip rotation ROM is the only kinematic parameter that showed a statistically significant difference between groups: DSA registered a significant reduction from PRE to T1 and T3; in addition, DSA had lower values at T1 (p = 0.04) and T3 (p = 0.04) compared with PL. ROM value for hip rotation, expressed in degrees, is the result of the difference between extra-rotation (max) and intra-rotation (min); therefore, the lower ROM value in T1 and T3 reported in the DSA group should be interpreted as a difference in the capacity of intra-rotating the hip in DSA compared with PL. The clinical interpretation of this kinematic parameter may indicate that the DSA approach, with consequent surgical sparing of the fascia lata, may result in higher hip stability at the immediate postoperative time points. This result is corroborated by the higher abduction/adduction ROM and the concomitant decrease of the hip rotation ROM, which is mainly ascribed to an improved intra-rotation component as reported in the literature (Ewen et al. 2012, Behery and Foucher 2014, Zeni et al. 2018, Yoo et al. 2019). Previous studies also report that kinematic data on healthy subjects indicates that during the abduction phase, corresponding to gait start, the hip is intra-rotated, supporting the importance of this kinematic parameter (Behery and Foucher 2014, Kolk et al. 2014).

The clinical results (HHS) as well as self-reported outcome measures (SF12, HOOS, VAS) were similar between the 2 groups. Surgical time was prolonged in the DSA group (90 vs. 77 minutes), possibly attributable to a prolonged learning curve on the new surgical access and the use of dedicated instruments for the DSA approach. We also found a reduction of intra- and perioperative blood loos in DSA compared with PL (149 vs. 216 mL), which we consider to be clinically important.

The number of serious adverse events was higher in DSA (3/25) compared with PL (1/25). In particular, 1 periprosthetic fracture of the greater trochanter and 2 anterior traumatic dislocations appeared in the DSA group. The periprosthetic fracture was treated with open reduction and internal fixation whereas, as concerns the 2 dislocations, cup inclination and anteversion values before reduction were 28° and 31° respectively, which may explain the instability. Both patients were treated with closed reduction and had no further consequences. The reported serious adverse events in DSA could be ascribed to the learning process for the new surgical approach, which implies reduced visualization of the proximal femur and acetabulum. Furthermore, 1 ischemic stroke occurred in the PL group.

The main strength of this study is the RCT design, which, together with appropriate statistically pre-determined sample size calculation, guarantees robustness of the findings. However, a possible limitation is the relatively short-term follow-up. Nevertheless, the present study focused on the short-term follow-up of patients (1–3 months) to investigate the potential effects on early functional recovery of this type of minimally invasive fascia lata sparing surgery.

In summary, we demonstrated that the novel direct superior approach is associated with a decrease in intra- and perioperative blood loss and with early improvements in TUG, spatiotemporal, and kinematic parameters highlighting rapid muscle strength recovery. Surgical time was longer in the DSA group. Clinical and self-reported functional scores did not differ between the 2 treatments.

## Supplementary Material

Supplemental Material

## Supplementary data

Table 2 and 3 are available as supplementary data in the online version of this article, http://dx.doi.org/10.1080/17453674.2020.1865633.
