# Supplementary material for: Direct superior approach versus posterolateral approach in total hip arthroplasty: a randomized controlled trial on early outcomes on gait, risk of fall, clinical and self-reported measurements
Source: Acta Orthop. 2021 Jan 7;92(3):274–9. doi: 10.1080/17453674.2020.1865633 (PMC8231353; doi:10.1080/17453674.2020.1865633)
Supplement: Supplemental Material [file IORT_A_1865633_SM4279.pdf]

Supplementary data

Table 2. Changes in risk of fall, TUG, and spatiotemporal parameters for direct superior approach (DSA) and posterolateral approach (PL) before (PRE), 1 month (T1), and 3 months after surgery (T3). Data are mean (SD)

Clinical variables	PRE	T1	T3	Effect of time p–value	Contrasts PRE vs T3 p-value	T1 vs T3 p-value
Risk of fall (OAK score 0–24)						
DAS	15 (6.1)	14 (7.4)	17 (5.6)	0.0001	0.06	0.03
PL	15 (4.9)	15 (6.1)	17 (5.8)		0.04	0.02
Timed Up and Go test (score 0–3)						
DAS	2.0 (0.89)	2.2 (0.48)	2.6 (0.50)	0.001	0.009	0.009
PL	1.9 (0.86)	2.0 (0.76)	2.3 (0.67)		0.2	0.1
Stance phase (%) surgical side						
DAS	65 (4.4)	66 (3.6)	65 (4.2)	0.1	1.0	0.2
PL	65 (4.7)	66 (4.4)	65 (4.6)		1.0	0.5
Stance phase (%) nonoperated side						
DAS	67 (6.2)	66 (3.9)	64 (3.7)	0.04	0.4	0.04
PL	65 (8.5)	67 (4.6)	65 (4.1)		0.5	< 0.001
Step length (m) surgical side						
DAS	0.42 (0.10)	0.42 (0.09)	0.47 (0.08)	< 0.001	0.09	0.001
PL	0.41 (0.09)	0.41 (0.07)	0.47 (0.08)		< 0.001	< 0.001
Step length (m) nonoperated side						
DAS	0.42 (0.08)	0.39 (0.09)	0.44 (0.09)	0.001	0.3	< 0.001
PL	0.41 (0.10)	0.39 (0.08)	0.45 (0.07)		0.3	0.001
Stride length (m)						
DAS	0.93 (0.20)	0.92 (0.20)	1.0 (0.18)	< 0.001	0.1	< 0.001
PL	0.92 (0.20)	0.90 (0.18)	1.1 (0.21)		0.002	0.004
Gait cadence (steps/min)						
DAS	95 (15)	94 (12)	101 (13)	< 0.001	0.2	< 0.001
PL	90 (18)	91 (14)	99 (14)		0.007	0.002
Gait Speed (m/s)						
DAS	0.60 (0.25)	0.56 (0.23)	0.73 (0.23)	< 0.001	0.09	< 0.001
PL	0.55 (0.22)	0.53 (0.22)	0.69 (0.23)		0.003	< 0.001

The table presents the results of mixed ANOVA. No significant differences found in interaction between group and time, the effect of group (DSA vs. PL), and the effect of time (PRE vs. T1).

 Acta Orthopaedica 2020; 91 (DOI: 10.1080/17453674.2020.1865633)

Table 3. Changes in kinematic parameters for direct superior approach (DSA) and posterolateral approach (PL) before (PRE), 1 month (T1), and 3 months after surgery (T3). Data are mean (SD)

Clinical variables	PRE	T1	T3	Interaction (group x time) p-value	Effect of time p-value	Contrasts PRE vs T1 p-value	PRE vs T3 p-value	T1 vs T3 p-value	Effect of group p-value
Hip flexion–extension ROM (°) surgical side									
DAS	26 (7.4)	26 (7.5)	29 (9.3)	0.3	< 0.001	1.0	0.08	0.3	0.2
PL	27 (7.6)	28 (5.5)	34 (5.4)			0.6	< 0.001	< 0.001	
Hip flexion–extension ROM (°) nonoperated side									
DAS	39 (8.3)	35 (6.6)	36 (9.8)	1.0	0.009	0.02	0.2	1.0	0.2
PL	38 (9.8)	35 (7.5)	38 (7.8)			0.05	1.0	0.002	
Hip abduction–adduction ROM (°) surgical side									
DAS	6.6 (1.9)	7.4 (2.8)	8.3 (2.9)	0.4	0.004	0.2	0.03	0.1	0.8
PL	6.6 (2.8)	6.7 (1.9)	7.7 (2.4)			1.0	0.2	0.03	
Hip abduction–adduction ROM (°) nonoperated side									
DAS	7.9 (2.0)	7.5 (2.1)	8.3 (2.6)	0.7	0.005	0.5	0.6	0.1	0.9
PL	7.5 (2.9)	7.5 (2.1)	8.5 (2.6)			1.0	0.4	0.03	
Hip rotation ROM (°) surgical side									
DAS	13 (3.6)	9.4 (2.6)	10 (3.6)	0.06	0.002	0.005	0.02	0.5	0.03 [a]
PL	13 (4.3)	12 (2.8)	13 (4.0)			0.5	1.0	0.7	
Hip rotation ROM (°) nonoperated side									
DAS	12 (3.7)	12 (4.5)	11 (4.8)	0.4	0.8	1.0	0.4	0.6	0.09
PL	14 (4.9)	14 (4.7)	14 (4.3)			0.9	0.8	1.0	
Hip obliquity ROM (°) surgical side									
DAS	4.4 (1.5)	5.3 (1.6)	4.8 (1.7)	0.002	0.7	0.008	0.5	0.3	0.8
PL	5.5 (1.9)	4.5 (1.3)	4.7 (1.3)			0.1	0.2	0.8	
Hip obliquity ROM (°) nonoperated side									
DAS	4.5 (1.6)	5.3 (1.6)	4.8 (1.9)	0.005	0.5	0.05	0.7	0.4	0.9
PL	5.6 (2.0)	4.6 (1.4)	4.6 (1.4)			0.09	0.1	1.0	

[a] T1: p = 0.04, T3: p = 0.04
The table presents the results of mixed ANOVA.
